# The "Clubs against Drugs" program in Stockholm, Sweden: two cross-sectional surveys examining drug use among staff at licensed premises

**DOI:** 10.1186/1747-597X-6-2

**Published:** 2011-02-07

**Authors:** Johanna Gripenberg Abdon, Eva Wallin, Sven Andréasson

**Affiliations:** 1 Department of Public Health Sciences, Karolinska Institutet, Stockholm, Sweden; 2 STAD, Stockholm Centre for Psychiatric Research and Education, Department of Clinical Neuroscience, Karolinska Institutet, Stockholm County Council Health Care Provision, Box 6031, 102 31 Stockholm, Sweden

## Abstract

**Background:**

The objective of this study is to examine self-reported drug use among staff at licensed premises, types of drugs used, attitudes towards drugs, and observed drug use among guests. Results are presented from two measurement points (in 2001 and 2007/08). This study was carried out within the framework of the "Clubs against Drugs" program, which is a community-based multi-component intervention targeting licensed premises in Stockholm, Sweden.

**Methods:**

Two cross-sectional surveys were conducted, the first in 2001 and the second in 2007/08. Staff at licensed premises attending server training were asked to participate in the anonymous survey. A survey was administered in a classroom setting and consisted of four sections: 1) demographics, 2) respondents' own drug use experience, 3) respondents' attitudes towards drug use, and 4) observed drug use among guests at licensed premises.

**Results:**

Data were collected from 446 staff in 2001 and 677 staff in 2007/08. The four most commonly used drugs among staff were cannabis, cocaine, amphetamine, and ecstasy. The highest rates of drug use were reported by staff in the two youngest age groups, i.e., those younger than 25 and those between the ages of 25 and 29. In 2007/08 staff reported significantly lower rates of drug use than staff in 2001. Last year drug use for the sample in 2007/08 was 19% compared to 27% for the 2001 sample. While drug-using staff compared to non drug-using staff reported more observations of drug use among guests, they were less inclined to intervene. Overall, staff reported restrictive attitudes towards drugs.

**Conclusions:**

The prevalence of life-time and last year drug use among staff at licensed premises is high compared to the general population in Sweden. Lower rates of self-reported drug use among staff were reported in 2007/08. The results of this study highlight that staff at licensed premises represent an important target population in club drug prevention programs.

## Background

During the last two decades there has been increased concern about the use of club drugs, (e.g., cocaine, amphetamine, ecstasy) in nightclubs around the world [1-4]. There are a number of public health issues related to illicit drug use such as unprotected sex, drugged driving, and both short- and long-term psychological and physical effects [2,5-8]. Studies from Sweden show that prices of illicit drugs have decreased some 40% to 60% and that the availability has increased [9]. Young people's attitudes towards drug use have become more liberal and they report that one of the most common places to find drugs is at licensed premises (e.g., clubs, bars) [10]. There has been a threefold increase in the number of licensed premises in Stockholm since the 1980s, from around 500 to 1600 today. This has resulted in an increased number of people being employed at licensed premises, as well as increased number of people visiting licensed premises as guests. Further in 1997, the allowed opening hours at licensed premises in Stockholm were extended to 5 a.m.. In reports from cities in other countries, extended opening hours have shown to increase the number of arrests and problem situations [11]. Swedish researchers have found that more than 10% of violent crimes are committed by people under the influence of illicit drugs [12].

Few studies on drug prevalence rates and attitudes with young adults and adults in Sweden exist, and most population surveys only include the use of cannabis [13]. As a result, there is a lack of appropriate comparison groups when studying club drug use at licensed premises. Nevertheless, school surveys are performed annually with 9^th ^and 11^th ^graders (15-17 years old). Here, the latest rates for ever-use of drugs reported by Swedish 15-year olds are 9% for boys and 7% for girls, while for 17-year olds the rates are 18% for boys and 15% for girls. These surveys reveal that Swedish adolescents have lower drug use rates compared to adolescents in most European countries and compared to the U.S. [14-16].

Since Sweden's strict drug laws aim at the reduction of drug use, rather than harm minimization, the focus of drug intervention strategies has historically been on primary prevention. In Sweden, all non-medical drug use is criminalized. Moreover, according to Swedish law licensed premises are not allowed to admit an obviously drug-intoxicated (alcohol or other drugs) person into their establishment. Therefore, if a guest is identified as obviously drug-intoxicated inside the premises, the guest has to be asked to leave. The responsibility to follow these laws lies with the owner and staff of licensed premises. For instance, at the entrance of licensed premises doormen have the responsibility to stop drug-intoxicated guests from entering. While inside the premises, the control of drug use shifts to the other staff members such as serving staff. Hence, all staff at licensed premises are an important target group to include in club drug interventions.

To our knowledge there are no published articles on the effectiveness of community-based prevention models for illicit club drug use targeting licensed premises. However, researchers are suggesting environmental changes and a healthy setting approach to nightclubs, as well as harm reduction strategies to minimize illicit club drug related problems [1,4,17,18]. On the other hand, research on alcohol prevention at licensed premises has been conducted in different settings and other countries [19-22]. In Stockholm, our research group at STAD (Stockholm prevents alcohol and drug problems), previously implemented and evaluated an alcohol intervention program for licensed premises, a so-called Responsible Beverage Service program (RBS). The RBS program in Stockholm resulted in a significant increase in the refusal rate of alcohol service to underage and intoxicated patrons, and a 29% decrease in police-reported violence [23-25].

Based on our experiences with alcohol prevention at licensed premises and the identified increased problems with illicit drug use at licensed premises, our research group initiated a new research program in 2001. This program "Clubs against Drugs", is a community-based multi-component intervention targeting licensed premises in Stockholm. The program (as well as the RBS program) is based on a systems approach to prevention with the aim at reducing drug use both among guest and staff at licensed premises [26,27]. Components of the program include community mobilization, policy work, increased enforcement, drug-training, changes in the physical environment at licensed premises, and media advocacy and public relations work.

Prevalence studies and needs assessment at project initiation showed that staff (mainly serving staff) at licensed premises in Stockholm use illicit drugs to a much higher extent than the general population [28,29]. An important focus of the "Clubs against Drugs" prevention program has therefore been to target staff to reduce the overall drug use at licensed premises by using a comprehensive drug policy approach. This included development of written policies, education to staff, training for managers, drug-testing, and rehabilitation [30,31]. Other important components of the policy work were information about the drug policy and the "Clubs against Drugs" intervention to all newly hired, as well as mandatory signing of the drug policy document by all staff. The policy document stated for example that drug use was not accepted among staff and guests, and that obviously drug-intoxicated guests should not be admitted into licensed premises.

A number of studies have been, and are currently being conducted to evaluate the effects of this intervention [32]. The objective of this study, however, is to examine self-reported drug use among staff at licensed premises, types of drugs used, attitudes towards drugs, and observed drug use among guests.

## Methods

### Design, Participants and Setting

Two surveys were distributed to staff at licensed premises during server trainings, the first in 2001, and the second in 2007/08. Both surveys were conducted during a one-year period, (from January 2001 to December 2001, and from April 2007 to March 2008). Survey participants included staff working at licensed premises in the central part of Stockholm. The majority of the participants attending server training were servers and bar staff, and not owners or doormen.

A non-randomized, cross-sectional survey design, conducted in a classroom setting, was employed for several reasons. Our earlier experiences with collecting data from this group (i.e., staff at licensed premises) using postal surveys resulted in very low response rates. Furthermore, in Sweden, there are no comprehensive registers of individuals employed at licensed premises, few are members of the unions, and staff turnover is high. Therefore, it was not feasible for us to randomly select survey participants. A cohort study could not be performed mainly due to perceived reluctance from staff to participate in a follow-up study, concerns about anonymity, and high staff turnover at licensed premises.

Since the year 2000, licensed premises in the Stockholm Municipality that are open after 1 a.m. are required to send their staff to a two-day server training. After staff has completed the training and passed the written exam they receive a diploma and are not required to attend server training again. The curriculum of the training has remained the same between the two measurement points. The server training focuses on responsible beverage service and only includes a short session on other club drugs. Today, an average of 700-800 staff participate in the 12 to 16 server trainings taking place on a yearly basis. We therefore make the assumption that individuals attending yearly server trainings are representative of staff working at licensed premises with late open hours in Stockholm.

This study has been approved by the Regional Ethical Review Board at the Karolinska Institutet in Stockholm (2005/1372-31).

### Survey Instrument

A survey was developed and reviewed by colleagues and community stakeholders, such as owners of licensed premises, before it was pilot tested. The survey included four sections. The first section collected demographic variables (gender, age, years working experience at licensed premises, location of workplace). The next section had questions on respondents' perception of drug prevalence at establishments in Stockholm (e.g., observed drug-intoxicated guests, drug offers, drug intake). This was followed by a section with questions on their own attitudes towards drugs (e.g., laws for licensed premises, drug laws, illicit vs. legal drugs). In the last section, participants were asked about their personal drug use experience (e.g., life-time use, last year use, types of drugs used).

### Procedure

During the server trainings all participants were asked to complete the anonymous survey. The purpose of the study was presented by the researchers and the participants were asked to reposition their chairs to ensure anonymity and confidentiality. When the survey was completed each participant placed it in an envelope, sealed it and then turned it in. The same procedures were used for both samples (2001 and 2007/08).

### Data analysis

Statistical analyses were carried out using the SAS 9.2 Software (SAS Institute Inc.) and PASW Statistics 18 software (SPSS Inc.). Comparisons between groups were performed using χ^2 ^tests. To evaluate interaction effects, logistic regression analysis with backward stepwise elimination of insignificant variables were used. Data at first measurement (2001) and at second measurement (2007/08) were treated independently with no identifying information. Thus, while respondents in the samples may overlap, they were treated as independent samples. However, it should be noted that overlaps are highly unlikely as staff are only required to attend server training once. Another statistical design issue is that staff may be nested within licensed premises. This design factor could not be incorporated into the analysis, since staff did not have to provide name of their workplace (due to anonymity reasons). Hence, the analysis conducted assumes independent observations. Only the participants that were employed at licensed premises in the central part of Stockholm were included in the analysis.

## Results

### Demographic characteristics of participants

At the first measurement in 2001, 446 participants completed the survey (Table 1). Sixty percent of the respondents were male. The mean age with the corresponding standard deviation (S.D.) was 29.1 years (S.D. = 8.6), and the participants had an average of 8.2 (S.D. = 6.8) years of work experience at licensed premises. The demographic characteristics of the 677 participants in 2007/08 were similar with a mean age of 28.8 (S.D. = 8.5), and mean years of working experience of 8.7 (S.D. = 7.3). There was no significant difference between the two measurements in mean age (p = 0.533) or mean years of working experience (p = 0.245). The only significant difference between the two sample groups was a decreased proportion of males at follow-up from 60% to 51% and hence an increased proportion of women (Table 1).

**Table 1:** Demographic comparison between 2001 and 2007/08 samples

Demographics	2001 (n = 446)	2007/08 (n = 677)	Significance χ^2 ^(d.f.), P
Sex (%)			
Male	59.8	51.3	
Female	40.2	48.7	7.75 (1), 0.005
Age (%)			
< 25 years	35.1	36.6	
25-29	26.2	29.3	
30-39	28.1	21.9	
40+	10.6	12.2	5.70 (3), 0.127

All participants attending the trainings agreed to complete the survey, giving a response rate of 100% both in 2001 and in 2007/08. However, there was an internal drop-out rate on some questions, with the highest being between 6-7% (both in 2001 and 2007/08) on the section on personal drug use experience. Only staff working at licensed premises in central Stockholm were included in the analysis. All 446 participants in 2001 worked at licensed premises in central Stockholm, and were therefore included in the study. In 2007/08, a total of 757 individuals agreed to participate, however, 80 of the respondents were excluded from the analysis since they were not working at a licensed premise in central Stockholm, resulting in a sample of 677 surveys. The majority of licensed premises that send their staff to trainings are the premises that remain open after 1 a.m. and therefore are obligated to train their staff. These types of licensed premises are often more focused on alcohol sales than food sales and are consequently categorized as higher risk venues.

### Self-reported rates of illicit drug use

Overall, 60% of the respondents reported ever-use of illicit drugs in 2001 compared to 53% in 2007/08 (Table 2). At first measurement, 27% had used a drug during the last year compared to 19% in 2007/08 (Table 3). The differences between the samples are statistically significant, with the greatest difference observed in reduction in rates of drug use last year for the second sample.

**Table 2:** Rates of reported drug use ever by age, 2001 compared to 2007/08

Age (years)	2001 (%)	2007/08 (%)	Significance^a^ χ^2 ^, P
< 25	68.5	56.9	5.12, 0.024
25-29	60.2	53.2	1.36, 0.244
30-39	61.5	50.7	3.00, 0.083
40+	27.9	44.7	3.28, 0.070
Total	60.3	52.7	6.08, 0.014

^a ^Chi-square tests were conducted, all with one degree of freedom.

**Table 3:** Rates of reported drug use last year by age, 2001 compared to 2007/08

Age (years)	2001 (%)	2007/08 (%)	Significance^a^ χ^2 ^, P
< 25	40.7	29.2	5.37, 0.020
25-29	32.8	18.0	8.50, 0.004
30-39	12.9	10.3	0.43, 0.511
40+	2.1	6.6	1.24, 0.266
Total	26.7	18.9	9.12, 0.003

^a ^Chi-square tests were conducted, all with one degree of freedom.

When comparing rates of reported drug use ever by age, the highest rates were found in the youngest age group both in 2001 and 2007/08 (Table 2). Significantly lower rates reported in 2007/08 applied only to participants in the youngest age group (< 25).

Table 3 shows rates of drug use last year by age for 2001 and 2007/08. Once again highest recent rates were reported by the <25 age group at both measurements. Here, the two youngest age groups reported significantly lower rates in last year drug use in the second sample (2007/08).

Table 4 lists rates of reported life-time and last year drug use by sex in 2001 and 2007/08. At both measurements, no significant differences were observed between males and females in rates of ever and recent use. In 2007/08 significantly lower rates were only observed among females' last year use (from 27% to 17%). A logistic regression analysis showed that females contribute significantly more than males to the overall lower rates of reported last year drug use. However, there was no interaction effect between sex and age.

**Table 4:** Rates of reported life-time and last year drug use by sex, 2001 compared to 2007/08

Sex	2001 (%)	2007/08 (%)	Significance^a^ χ^2 ^, P
Males ever-used	61.2	55.0	2.28, 0.131
Males last year	26.8	21.0	2.72, 0.099
Females ever-used	59.5	50.7	3.42, 0.064
Females last year	27.0	16.7	7.27, 0.007
Total ever-used	60.3	52.7	6.08, 0.014
Total last year	26.7	18.9	9.12, 0.003

^a ^Chi-square tests were conducted, all with one degree of freedom.

### Types of Drugs Used

Cannabis was the most widely used drug both in 2001 and 2007/08 among survey participants, followed by cocaine, amphetamine, and ecstasy (Table 5). The rates of reported ever-use for these four drugs were lower in 2007/08. Amphetamines were the only drug type that showed a significant lower rate in 2007/08, from 20% to 15%. Reported rates for heroin, LSD, and mushroom use were low, ranging from 0-7%. Other types of drugs, such as GHB and ketamine, were also reported but at extremely low rates (< 1%).

**Table 5:** Rates of reported ever-use of different drugs among staff 2001 compared to 2007/08

	2001 (%)	2007/08 (%)	Significance^a^ χ^2 ^, P
Cannabis	52.7	47.1	3.34, 0.068
Cocaine	24.9	21.9	1.39, 0.239
Amphetamine	20.4	14.5	6.75, 0.009
Ecstasy	16.4	13.2	2.26, 0.133
Heroin IV-use^b^	0.2	0.0	1.52, 0.397
Heroin inhalant^b^	0.9	1.6	1.08, 0.298
LSD	6.1	5.9	0.01, 0.920
Mushrooms	7.2	5.6	1.12, 0.289
Sedatives	14.0	14.5	0.05, 0.821

^a ^Chi-square tests were conducted, all with one degree of freedom.

^b ^Fisher's Exact Test was used since 2 cells have expected count less than 5.

### Behavioral intentions and attitudes towards drugs

Table 6 lists the rates of responses for different attitude questions. At first measurement in 2001, the majority of individuals supported Swedish drug laws requiring drug-intoxicated guests being asked to leave (74%). However, the participants in 2007/08 reported an even greater (85%) level of support for this law.

**Table 6:** Rates of responses for questions on attitude and behavioral intention, 2001 compared to 2007/08

	2001 (%)	2007/08 (%)	Significance^a^ χ^2 ^, P
% responding that drug-intoxicated guests always should be asked to leave licensed premises	73.9	84.6	19.03, <0.001
% responding that it should be illegal to be drug-intoxicated	62.4	67.4	2.87, 0.093
% responding that illicit drugs should be legal such as tobacco and alcohol	5.9	4.9	0.46, 0.496
% responding that they would call the police if they see someone take drugs at the licensed premises where they work	38.8	52.9	21.41, <0.001

^a ^Chi-square tests were conducted, all with one degree of freedom.

A higher rate of the 2007/08 sample, 53% compared to 38% of the 2001 sample, responded that they would call the police if they saw someone take drugs at the licensed premise where they worked. Participants reported little support for legalizing illicit drugs. Only 6% in 2001 and 5% in 2007/08 supported legalization of illicit drugs.

### Observed drug use among guests

In 2001, 83% of the participants observed drug-intoxicated guests at licensed premises in Stockholm during the last six months as compared to 76% of the participants in 2007/08 (Table 7). Furthermore, at the first measurement, 48% had observed a guest being offered a drug at licensed premises in Stockholm and 46% had observed a guest take a drug. In 2007/08, the participants observed fewer offers and intake of drugs with rates at 39% and 38% respectively. As shown in Table 7, the participants in the 2007/08 measurement reported significantly lower rates for all manner of observed drug use compared to the sample in 2001.

**Table 7:** Rates of observed drug-intoxicated guest, drug offer, and drug intake, 2001 compared to 2007/08

	2001 (%)	2007/08 (%)	Significance^a^ χ^2 ^, P
% observed drug-intoxicated guest last 6 months	82.8	75.9	7.73, 0.006
% observed drug offer	48.1	39.0	9.04, 0.003
% observed drug intake	46.2	37.9	7.58, 0.006
% responding there is more drugs today at licensed premises in Stockholm than 5 years ago	44.5	26.0	44.47, <0.001

^a ^Chi-square tests were conducted, all with one degree of freedom.

### Last year drug-users vs. non drug-users

In Table 8, staff that have used drugs during the last year are compared to staff that have never used drugs. The two groups are compared on observations of drug use at their workplace and their attitudes towards drug use and their behavioral intentions. There are significant differences between the two groups. Staff that are drug-users report higher rates of observed drug use among guests. They also have more liberal attitudes towards drug use than non drug-users. Further, drug-users are less likely to call the police if they see someone take a drug at the licensed premise where they work (drug-users 20% vs. non drug-users 81%).

**Table 8:** Comparisons between last year drug-users and non drug-users, (2001 and 2007/08 participants).

	Rates for last year drug-users (%) (n = 232)	Rates for non drug-users (%) (n = 474)	Significance^a^ χ^2 ^, P
% observed drug-intoxicated guest last 6 months	90.5	70.6	34.65, <0.001
% observed drug offer	65.5	19.9	67.5, <0.001
% responding that drug-intoxicated guests always should be asked to leave licensed premises	63.5	87.4	53.93, <0.001
% responding that it should be illegal to be drug-intoxicated	41.6	75.0	73.93, <0.001
% responding that they would call the police if they see someone take drugs at the licesed premises where they work	19.5	80.5	52.26, <0.001

^a ^Chi-square tests were conducted, all with one degree of freedom.

## Discussion

The presented results suggest that drug use is high among staff at licensed premises in Stockholm. However, staff in 2007/08 reported lower rates of self-reported drug use, both life-time use and more interestingly, last year use, than staff in 2001.

The 2001 sample and 2007/08 sample also reported different rates of behavioral intention and dissimilar attitudes towards drugs. A higher number of the 2007/08 sample responded that drug-intoxicated guests should always be asked to leave licensed premises. In addition, a significantly higher number in 2007/08 reported that they would call the police if they saw someone take drugs at the licensed premise where they worked. Finally, the staff in 2007/08 reported significantly lower rates of observed drug-intoxicated guests, observed drug offers and drug intake.

Comparisons among staff that are last year drug-users and non drug-users verify that drug-using staff observe more drug use among guests, have a more liberal attitude towards drug use, and are less likely to intervene than non drug-using staff. The data suggest that in order to be more effective in reducing drug use among guests at licensed premises, drug use among staff should be targeted as well. Hence, staff at licensed premises are an important population to target in club drug prevention programs.

Staff at licensed premises had a much higher life-time and last year prevalence of drug use as compared to the general population in Sweden. For example, in 2003, 17% of Swedish 16-24 year olds reported ever-use of illicit drugs [10] compared to 57% of staff in the same age group in our survey 2007/08. Possible explanations for high self-reported drug use among our surveyed staff may include environmental factors such as greater access to drugs at licensed premises than other workplaces, and stressful work shifts with late hours. Another possible explanation could be that individuals with sensation-seeking personality may be more attracted to work in the nightlife setting [33]. However, despite the fact that the study participants reported a high prevalence of drug use the majority supported Sweden's strict drug laws (Table 6).

It was reported that the four most commonly used drugs in 2001 and 2007/08 were cannabis, cocaine, amphetamine, and ecstasy (Table 4). The rates for these four drugs were lower in 2007/08. Specifically, amphetamine had decreased the most and cocaine the least. This is in accordance with reports from the Police Authority and Custom Control Department, showing that the availability of cocaine has increased and prices have decreased in Stockholm [34]. The most popular types of club drugs used can vary in different settings, cultures, and countries. Researchers from other countries report that other types of drugs, for instance ketamine and GHB, are being used as club drugs [35,36]. The participants of our study reported very low rates of these club drugs. Here we measured staffs' self-reported drug use and their observations of drug use at licensed premises. Other researchers have studied prevalence rates for drug use among guests using self-reports and biological assays. For example, in a Swiss study conducted at dance music events, the rate of attendees ever-use of ecstasy and cocaine was 40% and 36% respectively [2]. Furthermore, researchers in the US found that 25% of the guests in the club setting used illicit drugs [3].

The objective of this study was to examine self-reported drug use among staff at licensed premises, types of drugs used, attitudes towards drugs and observed drug use among guests, and not to study the effects of the "Clubs against Drugs" program. The nature of a multi-component intervention such as the "Clubs against Drugs" complicates the explanation of these findings. Nevertheless, in the absence of other reasons for the decrease in drug use, we propose that it might be possible that the "Clubs against Drugs" program may have contributed to this result. It should be kept in mind that the intervention program was quite extensive and that no competing activities have transpired with the targeted licensed premises during the intervention period. The intervention strategies were implemented immediately following the first measurement in 2001. The policy work component of the intervention focused the most on preventing club drug use among staff. In 2007, at the time for the second measurement, more than 150 owners and managers had been policy-trained and over 400 doormen had passed the two-day drug-training course.

There are limitations to this study that constitute possible threats to the validity of our findings. As mentioned earlier, the use of a non-randomized cross-sectional design reduces our ability to interpret the results causally. The absence of a control group and national comparison data are further limitations. However, it is important to note that when comparing the two sample groups (Table 1) they are very similar in most demographic characteristics.

Another concern to be addressed is whether or not the reported lower rates of drug use at licensed premises could be explained by displacement of problems. The reduction in reported drug use could partly be the result of staff that use drugs choose to work at licensed premises not involved in the drug prevention work, or in another city. Additionally, guests that use drugs may have chosen to go to other licensed premises. But, it seems unlikely that this is the main explanation for the results, as these licensed premises in downtown Stockholm are very popular workplaces as well as popular for guests to visit. A multi-component prevention program in Australia, also supports the idea that guests choose to stay at their favorite establishment even after the implementation of intervention programs [37].

There are also some strengths of this study. The response rate was very high, all of the staff attending server training agreed to participate. The highest internal drop-out rate, at both measurements, was approximately 6-7% for questions on self-reported drug use. The same procedures for sampling and data collection were used in 2001 and in 2007/08. Since both measurements were conducted during a one year period, seasonal variations of drug use would not explain the results. Even though random sampling was not possible, we would like to argue that the sample used is the best available representation of staff from licensed premises with late open hours in central Stockholm.

The majority of drug prevention activities are focused on school prevention programs. However, this study emphasizes the importance of also developing prevention programs within other arenas. The nightlife scene is a high-risk setting for club drug use. Previous results published by our research group, [32] as well as results presented herein, indicate that the intervention might have reduced the rates of drug use at licensed premises in Stockholm.

## Conclusions

Our data demonstrate that drug use among staff at licensed premises is high compared to the general population in Sweden. The high levels of reported drug use found in this study are therefore a cause for concern. However, staff at licensed premises reported lower rates of self-reported drug use and observed drug use in 2007/08 compared to 2001. There are significant differences between staff that use drugs and staff that do not. Drug-using staff reported observing more drug use among guests, but were less inclined to intervene.

This study underscores the importance of developing prevention programs within high-risk social settings. Our results highlight that the nightlife scene continues to be a high-risk setting for club drug use in Stockholm and that staff at licensed premises are an important target in club drug prevention programs. Further research is needed to study club drug use and to explore the potential of prevention programs in the club setting.

## Competing interests

The authors declare that they have no competing interests.

## Authors' contributions

All authors contributed to the design of the study. JGA collected and analyzed the data and wrote the manuscript. EW and SA assisted with data analyses and edited the manuscript. All authors read and approved the final manuscript.
